# Characteristics of carotid atherosclerotic plaques of chronic lipid apheresis patients as assessed by *In Vivo* High-Resolution CMR - a comparative analysis

**DOI:** 10.1186/1532-429X-14-80

**Published:** 2012-11-29

**Authors:** Jochen M Grimm, Konstantin Nikolaou, Andreas Schindler, Reinhard Hettich, Franz Heigl, Clemens C Cyran, Florian Schwarz, Reinhard Klingel, Anna Karpinska, Chun Yuan, Martin Dichgans, Maximilian F Reiser, Tobias Saam

**Affiliations:** 1 Institute for Clinical Radiology, Ludwig-Maximilians-University Hospital Munich, Pettenkoferstr. 8a, 80336, Munich, Germany; 2 Medizinisches Versorgungszentrum Kempten-Allgäu, Kempten-Allgäu, Germany; 3 Apheresis Research Institute, Cologne, Germany; 4 Department of Radiology, University of Washington School of Medicine, Seattle, USA; 5 Institute for Stroke and Dementia Research, Ludwig-Maximilians-University Hospital Munich, Munich, Germany

**Keywords:** Atherosclerosis, Lipid apheresis, Cardiovascular MR, Plaque imaging, Stroke, Cardiovascular disease

## Abstract

**Background:**

Components of carotid atherosclerotic plaques can reliably be identified and quantified using high resolution *in vivo* 3-Tesla CMR. It is suspected that lipid apheresis therapy in addition to lowering serum lipid levels also has an influence on development and progression of atherosclerotic plaques. The purpose of this study was to evaluate the influence of chronic lipid apheresis (LA) on the composition of atherosclerotic carotid plaques.

**Methods:**

32 arteries of 16 patients during chronic LA-therapy with carotid plaques and stenosis of 1–80% were matched according to degree of stenosis with 32 patients, who had recently suffered an ischemic stroke. Of these patients only the asymptomatic carotid artery was analyzed. All patients underwent black-blood 3 T CMR of the carotids using parallel imaging and dedicated surface coils. Cardiovascular risk factors were recorded. Morphology and composition of carotid plaques were evaluated. For statistical evaluation Fisher’s Exact and unpaired *t*-test were used. A p-value <0.05 was considered statistically significant.

**Results:**

Patients in the LA-group were younger (63.5 vs. 73.9. years, p<0.05), had a higher prevalence of hypercholesterolemia and of established coronary heart disease in patients and in first-degree relatives (p<0.05, respectively). LA-patients had smaller maximum wall areas (49.7 vs. 59.6mm^2^, p<0.05), showed lower prevalence of lipid cores (28.1% vs. 56.3%, p<0.05) and the lipid content was smaller than in the control group (5.0 vs. 11.6%, p<0.05). Minimum lumen areas and maximum total vessel areas did not differ significantly between both groups.

**Conclusion:**

Results of this study suggest that, despite a severer risk profile for cardiovascular complications in LA-patients, chronic LA is associated with significantly lower lipid content in carotid plaques compared to plaques of patients without LA with similar degrees of stenosis, which is characteristic of clinically stable plaques.

## Background

Cardiovascular events, such as myocardial infarction or stroke are the leading cause of death in the western world. The incidence of cardiovascular diseases like ischemic stroke or infarction of heart muscle tissue is well correlated with a number of risk factors. Dyslipidemia is one of the most important established risk factors being causally related to cardiovascular events [1].

It has been shown that lowering of pathologically high serum levels of low density lipoprotein cholesterol (LDL-C) either by pharmacotherapy or lipid apheresis therapy significantly reduces cardiovascular morbidity and mortality [2,3]. However, how exactly this positive effect is achieved on a pathophysiological level is not completely understood.

One possible explanation is the improvement of rheological properties of the blood after lipid lowering therapy [4]. High levels of LDL-C have also been associated with increased inflammatory activity in vessel walls and reduced vasodilatory activity [5,6]. It has also been suspected that lipid lowering therapy in dyslipidemic patients leads to regression of atherosclerotic disease and reduction of the lipid component of atherosclerotic plaques [7,8].

Large vessel atherosclerotic disease is considered responsible for 20–30% of ischemic strokes [9]. It has been shown that cardiovascular events are correlated not only with the degree of stenosis of the respective vessel but even more so with the properties of the stenosing plaque [10], which promoted the concept of the complicated or vulnerable plaque [11,12]. One of the key features of the so-called vulnerable plaques is the large lipid/necrotic core covered by a thin fibrous cap [11,12]. This leads to the hypothesis that a decrease in their lipid content stabilizes atherosclerotic plaques.

Lipid apheresis therapy is an effective therapeutic option to lower serum lipid levels in patients with extensive hyperlipidemia, where medication alone is insufficient to reduce serum lipids to target levels [13]. A decrease of the size of atherosclerotic plaques and possible beneficial change of their composition have been reported in dyslipidemic patients under lipid lowering therapy [7,14,15]. It has been suspected that dyslipidemia is associated with an increased prevalence of soft plaques [16]. Consequently, a decrease in serum lipid levels should have an influence on prevalence of soft plaques and plaque composition [17] and may lower the fraction of vulnerable plaques among atherosclerotic lesions [8,18]. This mechanism should as well be relevant in lipid apheresis possibly caused by a direct lowering effect on the lipid content of atherosclerotic plaques [19]. This might offer an additional explanation for the observed decrease of cardiovascular events during chronic lipid apheresis.

A number of techniques allow for the assessment of atherosclerotic disease, among these extra- and intravascular ultrasound, CT-angiography, conventional angiography and cardiovascular magnetic resonance (CMR) plaque imaging. CMR plaque imaging has become one of the most reliable techniques to assess atherosclerotic plaque morphology and composition in good correlation with histopathology [20,21]. It is non-invasive, involves no ionizing radiation and is well suited to reliably detect and quantify plaque components including the lipid content of atherosclerotic plaques [22].

Existing studies have evaluated the effect of lipid lowering pharmacotherapy on atherosclerotic plaque development using CMR [7] or the effect of lipid apheresis on coronary plaque composition using ultrasound [23]. Other studies evaluated coronary plaque composition in relation to serum lipid levels using computed tomography angiography [17]. One Study evaluated the development of aortic atherosclerotic plaques in relation to serum lipid levels in rabbits using CMR [24]. To our knowledge, no studies have evaluated the effect of chronic lipid apheresis on carotid atherosclerotic plaque composition using CMR plaque imaging in human patients.

The purpose of this prospective comparative cross sectional study was to evaluate the influence of chronic lipid apheresis (LA) on the composition of atherosclerotic carotid plaques using high resolution *in vivo* 3-Tesla CMR with parallel imaging techniques.

## Methods

### Patient selection

A total of 48 patients were enrolled in this prospective cross sectional, monocentric observational study, which has been approved by the local institutional ethics committee. In the chronic lipid apheresis group we examined 32 arteries of 16 patients who had been treated by chronic lipid apheresis for more than 5 years (7 patients by heparin mediated plasma precipitation in acidic milieu (HELP ©), 7 patients by temperature optimized double-filtration plasmapheresis (Lipidfiltration ©), 2 patients by polyacrylat full blood adsorption (DALI ©)). Indication for chronic lipid apheresis was severe therapy refractory hypercholesterolemia (N=13) or isolated Lp(a) hyperlipoproteinemia with progressive vascular disease (N=3). Lipid apheresis treatment had been performed in intervals between 3 and 14 days. In the control group we included 32 consecutive patients matched for the degree of vascular stenosis, who had recently suffered an ischemic stroke. Of these patients only the asymptomatic artery contralateral to the ischemic stroke was included. The majority of patients from the lipid apheresis group (9 out of 16, 56%) and control group (20 out of 32, 63%) received lipid lowering medication at the time of examination. Manifest coronary artery disease and cardiovascular risk factors including serum lipid levels, arterial hypertension, active or past nicotine abuse, diabetes mellitus, family history of coronary artery disease and body mass index (BMI) were recorded. Values for LDL and Lp (a) for the lipid apheresis group are given as mean levels calculated as proposed by Kroon et al. [25]. Other serum lipid levels for the lipid apheresis group are given as average of measurements before and after apheresis therapy. Serum lipid levels for the control group were measured at the time of the CMR examination performed at time of admission due to acute ischemic stroke.

### MR imaging protocol

All subjects underwent a carotid black-blood high resolution CMR at 3.0-Tesla on a Siemens Verio Scanner (Siemens AG, Erlangen, Germany) using surface coils (Machnet, Elde, Netherlands) and a previously published multi-sequence protocol [26] (time-of-flight MR angiography (TOF), axial fat suppressed pre- and post- contrast black-blood T1-, PD- and T2- weighted sequences; best in-plane resolution 0.5 × 0.5 mm^2^). Parallel imaging based on the generalized autocalibrating partially parallel acquisition (GRAPPA) algorithm was used for all sequences with a parallel acquisition technique (PAT) acceleration factor of 2. Total imaging time was 17:43 minutes. Gadobutrol (Gadovist®, Bayer Schering, Leverkusen, Germany) of 0.1 mmol/kg (0.1 ml/kg) was given at a rate of 3 ml/s. Post-contrast T1w imaging was performed approximately 5 minutes after intravenous injection of the contrast agent. Each scan covered 30 mm (2 mm slice thickness × 15 matched images across the 5 sequences).

### Image analysis

An image-quality rating (4-point scale, 1 = non-diagnostic, 2 = poor, 3 = good, 4 = excellent) was assigned to all MR images. MR data were then independently reviewed by two experienced radiologists (T.S. and J.G.) who where blinded regarding the clinical status of the patient. In case of discrepancy, a consensus decision was made. Atherosclerotic plaques in the carotid arteries were recorded and classified according to the modified criteria of the American Heart Association [27]. For definition of a complicated AHA-LT6 plaque, at least one of the following three criteria was required: fibrous cap rupture, intraplaque hemorrhage, juxtaluminal hemorrhage/mural thrombus. Area measurements of the lumen, outer wall, and tissue components were obtained using a custom-designed image analysis tool CASCADE (University of Washington, Seattle, USA). Tissue components (lipid-rich necrotic core, calcification, hemorrhage) were identified based on previously published criteria [20]. The “total vessel area” included the lumen and wall areas. The normalized wall index (NWI) was calculated by dividing the wall area by the total vessel area.

### Statistical analysis

For statistical comparison of the patients with and without lipid apheresis therapy, the Mann–Whitney test with correction for ties was used for variables describing frequencies of plaque features, which had skewed distribution. For continuous variables, the Student unpaired *t*-test with equal or unequal variances (as appropriate) was used. To compare clinical demographics between both groups, Fisher’s exact test was used for categorical variables, and the unpaired *t*-test was used for continuous variables. Categorical variables are presented as absolute and relative frequencies, while continuous variables are presented as mean and standard deviation. Tissue components were calculated as percentages of the vessel wall. Analyses were carried out in SPSS for Windows (version 14.0, IBM, Chicago, IL). A p-value of <0.05 was considered as statistically significant.

## Results

### Clinical data

Patients in the lipid apheresis group were younger (63.5 vs. 73.9 years, p<0.05) and had a higher prevalence of hypercholesterolemia (100% vs. 53%), coronary artery disease and/or family history of coronary artery disease (100% vs. 34%) (all p-values <0.05). Total cholesterol (148.8 vs. 200.8 mg/dl) and LDL (94.7 vs. 128.3 mg/dl) - averaged as described above - were significantly lower in the lipid apheresis group than in the control group (p<0.001). Consequently, LDL/HDL was also lower in the lipid apheresis group (2.0 vs. 2.8; p<0.01). No further statistically significant differences in clinical data were found [Table 1].

**Table 1:** Cinical data

**Parameter**	**Lipid apheresis group**	**Control group**	p-value*
	**(n=16 patients)**	**(n=32 patients)**	
Age ± 1 SD [years]	63.5 ± 9,1	73.9 ± 9.2	<0.05
Male [%]	68,8%	84.4%	ns
Body Mass Index	26.5 ± 4.4	25.1 ± 5.3	ns
Established CAD [%]	100%	21.9%	<0.001
**Risk Factors**			
Hypercholesterolemia [%]	100%	53.3%	0.008
Arterial Hypertension, [%]	75%	68.8%	ns
Active Nicotine Abuse [%]	6.3%	28.1%	ns
Former Nicotine Abuse [%]	50%	40.7%	ns
Diabetes Mellitus [%]	18.8%	21.9%	ns
Family History of CAD or CVD [%]	68.8%	34.4%	0.03
**Serum Lipids**			
Total Cholesterol [mg/dl]	148.8 ± 37.7	200.8 ± 47.1	<0.001
Triglycerides [mg/dl]	139.1 ± 93.9	142.0 ± 73.5	ns
LDL [mg/dl]	94.7 ± 28.9	128.3 ± 35.8	<0.001
VLDL [mg/dl]	18.6 ± 7.9	22.4 ± 10.4	ns
HDL [mg/dl]	49.3 ± 12.7	48.2 ± 9.2	ns
LDL/HDL	2.0 ± 1.0	2.8 ± 0.8	0.008
Lipoprotein (a) [mg/dl]	59.9 ± 66.6	47.4 ± 49.4	ns

Two-sided Fisher’s Exact Test or unpaired t-test; SD=Standard Deviation; CAD=Coronary Artery Disease; CVD=Cerebrovascular Disease (TIA or Stroke). Values for LDL and Lp (a) for the lipid apheresis group are given as mean levels calculated as proposed by Kroon et al. [25]. Other serum lipid levels for the lipid apheresis group are given as average of measurements before and after apheresis therapy.

### Imaging data

#### Image quality

**Figure 1:**
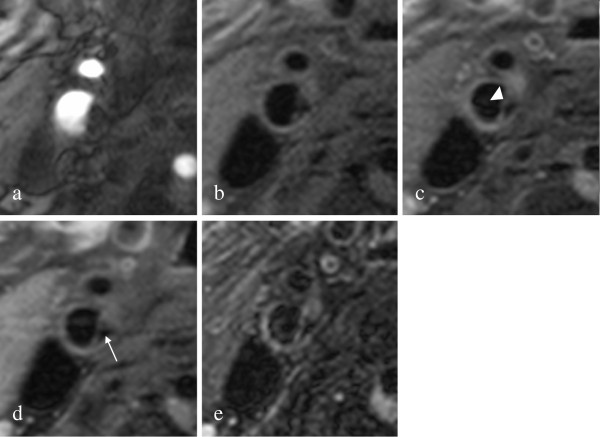
**Imaging example Lipid Apheresis Group. **shows axial TOF (**a**), T1w without (**b**) and with (**c**) contrast agent, PD (**d**) and T2w (**e**) MR-images of an American Heart Association Type II plaque in the right internal carotid artery of a patient from the lipid apheresis group. This plaque is mainly calcified (arrow); no lipid necrotic core can be seen. The fibrous cap appears intact (arrowhead).

**Figure 2:**
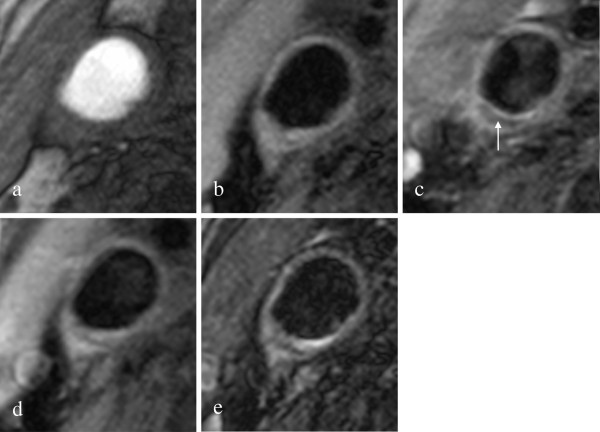
**Imaging Example Control Group. **shows an American Heart Association type V plaque at the bifurcation in the right internal carotid artery of a patient from the control group in axial TOF (**a**), T1w without (**b**) and with (**c**) contrast agent, PDw (**d**) and T2w (**e**) MR-images. The plaque consists mainly of a necrotic lipid core without contrast enhancement (arrow) and no relevant calcification.

48 out of 50 scans (96.0%) had a sufficient image quality (image quality ≥ 2), with an average image quality rating of 3.6 for the lipid apheresis patients and 3.4 for the control group (p = ns). The two excluded scans were scans of the control group. All data are given for 16 lipid apheresis patients and 32 patients of the control group. Imaging examples are given [Figures 1 and 2].

#### Qualitative and quantitative plaque imaging characteristics

Patients in the lipid apheresis group showed a lower prevalence of lipid cores compared to the control subjects (28.1 vs. 56.3%; p<0.05). The maximum lipid content as percentage of the vessel wall was also smaller in the lipid apheresis group (5.0 vs. 11.6%, p<0.05). There was a tendency towards larger minimum lumen areas in the lipid apheresis group compared to the control group, however, both parameters did not differ significantly between the two groups (15.9 vs. 13.0 mm^2^, p=ns). The normalized wall index and the maximum wall area were smaller in the lipid apheresis group than in the control group (0.61 vs. 0.72; p<0.001 and 49.7 vs. 59.6 mm^2^; p<0.05). Prevalence of plaque calcifications and calcification content did not differ between both groups. Intraplaque hemorrhage prevalence and hemorrhage content did not differ significantly between both groups (9.4% vs. 15.6% and 2.1 vs. 2.3%; p=ns), although there was a tendency towards a higher prevalence of hemorrhages in the control group [Table 2].

**Table 2:** MR-Imaging data

	**Lipid apheresis group**	**Control group**	p-value*
	**(n=32 arteries)**	**(n=32 arteries)**	
Lumen Area	15.9 ± 6.3	13.0 ± 7.6	ns
[mm^2^, Minimum]			
Wall Area	49.7 ± 11.8	59.6 ± 21.6	0.03
[mm^2^, Maximum]			
Normalized Wall Index	0.61 ± 0.09	0.72 ± 0.12	<0.001
[Maximum]			
Outer Wall Area	105.4 ± 28.3	102.0 ± 35.4	ns
[mm^2^, Maximum]			
**Plaque Components (quantitative)**			
Necrotic Lipid Core	5.0 ± 9.4	11.6 ± 13.4	0.025
[% of Vessel Wall, Maximum]			
Intraplaque Hemorrhage	2.1 ± 8.8	2.3 ± 6.8	ns
[% of Vessel Wall, Maximum]			
Calcifications	4.8 ± 6.0	5.3 ± 6.1	ns
[% of Vessel Wall, Maximum]			
**Prevalence of Plaque Components**			
Necrotic Lipid Core [%]	28.1	56.3	0.025
Intraplaque Hemorrhage [%]	9.4	15.6	ns
Calcifications [%]	53.1	56.3	ns
**American Heart Association (AHA) Lesion Type**			
Type I [%]	15.6	12.5	ns
Type III [%]	25.0	15.6	ns
Type IV/V [%]	13.0	37.5	0.023
Type VI [%]	9.4	15.6	ns
Type VII [%]	37.5	18.8	ns
Type VIII [%]	0	0	ns

* Unpaired t-test; SD=Standard Deviation.

#### AHA lesion type distribution

**Figure 3:**
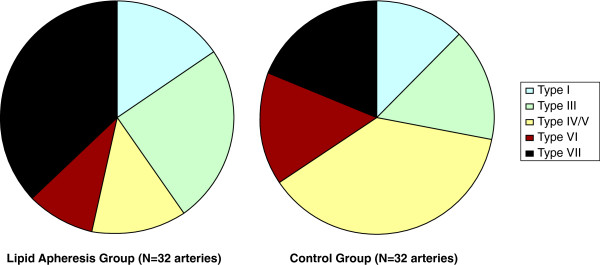
**AHA Lesion Type Distribution. **Visualizes the distribution of American Heart Association (AHA) lesion Types in the Lipid Apheresis and the Control Group. Note the higher prevalence of lipid rich type IV/V plaques in the Control Group. There also was a tendency toward higher prevalence of calcified Type VII plaques in the Lipid Apheresis Group.

AHA lesion type IV/V, which is characterized by the presence of a lipid core covered by a fibrous cap, was found significantly more often in the control group compared to the lipid apheresis group (38% vs. 13%; p<0.05). No other significant differences were found although there was a tendency towards a higher prevalence of calcified AHA type VII lesions in the lipid apheresis group compared to the control group (38% vs. 19%; p=ns) [Figure 3].

## Discussion

This cross-sectional study showed that patients who had been treated by chronic lipid apheresis therapy for more than 5 years had a significantly lower prevalence of lipid-rich plaques and lower plaque lipid content compared to asymptomatic arteries with similar degrees of stenosis. Patients on chronic lipid apheresis therapy had a lower wall area and a tendency towards more calcified plaques compared to the control group. Accordingly, total cholesterol, LDL and LDL/HDL were lower in the lipid apheresis group suggesting an influence of these parameters on plaque characteristics. Plaques of patients during chronic lipid apheresis showed characteristics of more stable plaques compared to the control group. This study also demonstrated significant differences in AHA lesion type distribution. AHA lesion type IV/V, characterized by a lipid core covered by a fibrous cap was significantly more common in the control group, whereas the relatively stable calcified AHA lesion type VII had a higher prevalence in the lipid apheresis group. In summary, these findings suggest a beneficial influence of lipid apheresis therapy on plaque composition despite the significantly severer risk profile in the LA-group considering the higher prevalence of hypercholesterolemia and positive family history of cardiovascular disease in LA-patients.

This result is in accordance with previously published studies, which suspected a decrease in the lipid component of atherosclerotic plaques of patients under lipid lowering therapy [17,19]. Underhill et al. [23] showed in a double-blind trial using 1.5-T CMR to image carotid atherosclerotic plaques at baseline and after 24 months of rosuvastatin treatment a significant reduction in lipid rich necrotic core, whereas the overall plaque burden remained unchanged over the course of 2 years of treatment. The authors concluded that statin therapy may have a beneficial effect on plaque volume and composition, as assessed by non-invasive CMR. Results of this study support the proposition that serum lipid levels correlate with lipid content of atherosclerotic lesions. Since lipid rich plaques seem to be a more vulnerable subgroup of atherosclerotic lesions, a decrease of their lipid component might help explain the decrease in cardiovascular incidents [8].

The minimal lumen area as an indirect measurement of stenosis was similar in both groups. We found smaller maximum wall areas in the lipid apheresis group and smaller normalized wall indices, suggesting a lower plaque burden in this group of patients. This might in part be explained by previous studies which have not found a significant increase in vessel lumen area of treated dyslipidemic patients over time while showing a significant decrease in vessel wall area [7].

Although the number of patients enrolled was relatively small, significant differences in atherosclerotic disease between lipid apheresis patients and the control group were identified. This study suggests that CMR is a powerful tool to noninvasively evaluate atherosclerotic disease systematically across different groups of patients and populations.

This study achieved diagnostic image quality in 48 out of 50 scans, demonstrating that this protocol can be successfully used in a clinical setting. This drop-out rate of only 4% is substantially lower than the drop-out rates reported in recent 1.5 T MR studies, in which up to 33% of all patients were excluded due to insufficient image quality [28,29].

### Limitations

Although this study shows significant differences in plaque morphology and lesion type distribution between the two groups, the number of patients in this study is relatively small, and a larger study performed in multiple centers is necessary to confirm these preliminary findings. Furthermore, the cross-sectional design of the study allows by definition only a snapshot of plaque burden and composition at one single time point and no definite conclusions can be drawn on the evolution of plaque composition over time. Only CMR examinations with at least average image quality were considered for the review, resulting in exclusion of two arteries from analysis. However, this exclusion rate is lower than in previously published studies [28,29] and should not have hampered the results. These results were based on a single imaging modality: CMR. Information gathered from other well-known imaging techniques, notably measurements of calcifications from computed tomography, might further illuminate the differences. However, CMR is generally considered best suited to identify and quantify atherosclerotic plaque components in the carotid arteries, shows good correlation with histology for quantitative measurements, and does not use ionizing radiation. Given these advantages and the comparative complexity of a multi-modality study, sole use of CMR was the logical choice for this study.

## Conclusion

Findings of this study suggest that chronic lipid apheresis therapy is associated with lower cholesterol, LDL and LDL/HDL as well as a markedly decreased lipid content in atherosclerotic carotid plaques as assessed by *in vivo* CMR as compared to a control group with similar degree of arterial stenosis. These findings suggest an influence of cholesterol, LDL and LDL/HDL on plaque characteristics as well as a stabilizing effect of chronic lipid apheresis therapy on carotid plaque composition and might explain the observed beneficial influence of lipid apheresis therapy on clinical outcome. In the future, prospective longitudinal studies are needed to confirm these promising results and to better assess individual plaque development over time during chronic lipid apheresis.

## Competing interests

The study was sponsored by Diamed.

## Authors’ contributions

JMG participated in data acquisition, statistical evaluation, drafted and revised the manuscript. KN participated in designing the study and revising the manuscript. AS participated in patient and data acquisition. RH participated in designing the study and revising the manuscript. FH participated in designing the study and revising the manuscript. CCC participated in patient and data acquisition. FS participated in patient and data acquisition. RK participated in designing the study and revising the manuscript. AK participated in evaluation of the data. CY participated in evaluating the data and revising the manuscript. MD participated in designing the study, evaluating the data and revising the manuscript. MFR participated in designing the study and revising the manuscript. TS participated in designing the study, acquiring and evaluating the data, and revising the manuscript. All authors read and approved the final manuscript.
